# Ag–Au Core–Shell Triangular Nanoprisms for Improving p-g-C_3_N_4_ Photocatalytic Hydrogen Production

**DOI:** 10.3390/nano11123347

**Published:** 2021-12-10

**Authors:** Yali Guo, Anzhou Xu, Juan Hou, Qingcui Liu, Hailong Li, Xuhong Guo

**Affiliations:** 1 Key Laboratory of Oasis Town and Mountain-Basin System Ecology of Xinjiang Bingtuan, College of Science, Shihezi University, Shihezi 832003, China; yaly9512@sina.com (Y.G.); xuanzhou012@163.com (A.X.); hjuan05@sina.com (J.H.); 2 Key Laboratory for Green Process of Chemical Engineering of Xinjiang Bingtuan, School of Chemistry and Chemical Engineering, Shihezi University, Shihezi 832003, China; 3 State Key Laboratory of Chemistry and Utilization of Carbon Based Energy Resources, Key Laboratory of Advanced Functional Materials, Autonomous Region, Institute of Applied Chemistry, College of Chemistry, Xinjiang University, Urumqi 830046, China; 20180331@163.com; 4 School of Chemical Engineering, East China University of Science and Technology, 130 Meilong Road, Shanghai 200237, China

**Keywords:** photocatalytic water splitting, bimetallic Ag@Au TNPs, plasmon effect, galvanic-free replacement

## Abstract

Ag–Au core–shell triangular nanoprisms (Ag@Au TNPs) have aroused extensive research interest in the field of hydrogen evolution reaction (HER) due to their strong plasmon effect and stability. Here, Ag@Au TNPs were fabricated by the galvanic-free replacement method. Then, we loaded them on protonated g-C_3_N_4_ nanoprisms (P–CN) by the electrostatic self-assembly method as an efficient plasmonic photocatalyst for HER. The hydrogen production rate of Ag@Au TNPs/P–CN (4.52 mmol/g/h) is 4.1 times higher than that of P–CN (1.11 mmol/g/h) under simulated sunlight irradiation, making it the most competitive material for water splitting. The formed Schottky junction helps to trap the hot electrons generated from Ag@Au TNPs, and the well-preserved tips of the Ag@Au TNPs can effectively generate an electromagnetic field to inhibit the photogenerated electron–holes pairs recombination. This study suggests that the rational design of Ag@Au TNPs by the galvanic-free replacement method is an effective co-catalyst for HER and boosting the additional combination of plasmonic metals and catalyst metals for the enhancement to HER.

## 1. Introduction

Photocatalysis using semiconductors is a promising method to alleviate energy crisis and environmental pollution [1]. Among many semiconductor photocatalysts, g-C_3_N_4_ is a kind of two-dimensional layered nonmetallic semiconductor with excellent hydrogen evolution reaction (HER) performance [2]. Nevertheless, the narrow absorption spectra and the easy recombination of photogenerated electron–hole pairs in pure g-C_3_N_4_ greatly reduce the efficiency of photocatalytic hydrogen production [3]. Choosing a plasmonic metal as co-catalyst to expand light absorption [4] or catalysis metal to trap electrons can solve the above problems. For example, Xue et al. used bimetallic a Au and Pt NPs co-decorated g-C_3_N_4_ plasmonic photocatalyst to demonstrate that the surface plasmon resonance (SPR) effect of Au and electron-sink function of Pt nanoparticles could improve the optical absorption property as well as photogenerated charge carriers separation of g-C_3_N_4_, synergistically facilitating the photocatalysis process [5].

As is known, Ag and Au have unique optical properties, particularly the localized surface plasmon resonance (LSPR) [6]. When they come into contact with a semiconductor, a metal–semiconductor Schottky junction will be formed to trap electrons [7]. Ag is probably the most important material in plasmonics, and it is able to support a strong surface plasmon across the spectrum from 300 to 1200 nm [8]. However, the pure Ag nanostructure is easy to be oxidized, leading to tip truncation and thus decreasing the enhancement [9]. Au, the most widely used plasmonic metal, has gained considerable attention owing to its superior stability confronting oxidization [10]. It is reasonable to mix Ag and Au at the atomic scale for combining the excellent plasmon resonance effect of Ag nanoparticles and the good chemical stability of Au nanoparticles, further realizing the application in an actual complex chemical environment [11]. Meanwhile, LSPR properties are also affected by different geometrical properties of metal nanostructures [12]. Spheres, nanocubes [13], and triangular nanoprisms [14] have very strong dipole resonance peaks that dominate the spectra. The wavelength of triangular nanoprisms red-shifts 200 nm relative to the nanocubes from 450 to 650 nm [15], while the wavelength of nanocubes red-shifts 50 nm relative to the spheres from 400 to 450 nm. In order to use the visible light in the solar spectrum more reasonably and effectively, it is highly desirable to choose the triangular nanoprisms [16]. The sharp corners of nanocubes and triangles allow dipole oscillation, which can inhibit charge recombination, thus reducing the restoring force, to produce a more resonance wavelength. Compared with nanocubes, triangular nanoprisms have sharper corners, which benefit the hot electrons collection. The production of electromagnetic field enhancements can drastically inhibit the recombination of hot electrons–holes at these sites for HER. Therefore, by depositing a conformal, thin shell of Au on the surface of Ag nanostructures, one would create Ag@Au triangular nanoprisms with excellence in both chemical stability and plasmonic activity.

Due to the different reduction potential between Ag and Au [17], when Ag nanostructures encounter HAuCl_4_, they generally produce porous structures such as nanoboxes [17] or nanoframes [18], which will cause holes to destroy the integrity of the morphology, negatively influencing their functional properties. Previously, Dong et al. synthesized Au shells with three and six atom layers thickness deposited on Ag nanocubes by increasing the pH of the system to inhibit the galvanic replacement reaction [13].

Inspired by the above considerations, we use a galvanic-free exchange reaction to synthesize Ag@Au TNPs, depositing them on the P–CN with an electrostatic self-assembly method. The beneficial effect of the Au shell not only strengthens the stability of the Ag core but also contributes to the HER. The formed Schottky junction helps to trap the hot electrons in the conduction band of the semiconductor by delaying electron transmission back to the plasmonic metal. The retention of triangular tips helps to gather the hot electrons and generate an electromagnetic field to inhibit the recombination of electron–hole pairs. All factors will contribute to a significant increase in the photocatalytic HER activity under the irradiation of simulated solar. The present work could provide alternative thoughts for plasmonic photocatalysts, which is also an efficient strategy for combining the plasmonic metals and catalysis metals for HER.

## 2. Materials and Methods

### 2.1. Chemicals

Silver nitrate (AgNO_3_, 99.99%) and sodium borohydride (NaBH_4_, 98.00%) were purchased from Sigma-Aldrich (Shanghai, China). Sodium citrate (C_6_H_5_Na_3_O_7_, 98.00%), urea, and hydrogen tetrachloroaurate hydrate (HAuCl_4_·4H_2_O) were purchased from Shanghai Sinopharm Chemical reagent. All chemicals were used as received without further treatment.

### 2.2. Synthesis of Ag@Au TNPs

In this paper, the triangular silver nanoprisms were prepared by the solvent reduction method [15]. Usually, the total volume of the reaction solution is fixed at 25.00 mL. In 24.14 mL of pure water, combine the aqueous solutions of AgNO_3_ (0.05 M, 50 µL), trisodium citrate (75 mM, 0.5 mL), and H_2_O_2_ (30 wt %, 60 µL), and stir vigorously at room temperature in air atmosphere. Sodium borohydride (NaBH_4_, 100 mM, 250 µL) was quickly injected into the mixture to obtain nanoprisms. After about 3 min, the colloid turned dark yellow due to the formation of small silver nanoparticles. In the next few minutes, the morphology continued to change from nanoparticles to nanoprisms, and the color of the solution changed to gray, green, yellow, red, purple, and blue. After the reaction, it was centrifuged at 10,000 rpm, and the supernatant was removed and stored in the refrigerator. After that, we use a galvanic-free exchange method deposit Au on the surface of triangular silver nanoprisms to fabricate Ag@Au TNPs [13]. By the standard synthesis, 2 mL of PVP (1 mM) solution was introduced into a 20 mL glass vial, and then, 0.5 mL of ascorbic acid (AA, 10 mM), 0.1 mL of aqueous NaOH (200 mM), and 1.7 mL suspension of Ag triangular nanoprisms (6.6 mg/mL) were added under magnetic stirring. Next, 0.4 mL of HAuCl_4_ aqueous solution (0.1 mM) was titrated into the mixture at a rate of 0.02 mL/min. After reacting for 10 min, the product was collected by centrifugation at 10,000 rpm and washed with deionized water for four times, and then submitted to TEM characterization.

### 2.3. Synthesis of Ag@Au TNPs/P–CN

The g-C_3_N_4_ was synthesized from dicyandiamide and urea [1]. First, add 4 g of dicyandiamide and 6 g of urea to the mortar, grind them for half an hour until they are mixed well, put them in a muffle furnace at a temperature increase rate of 5 °C/min to 550 °C for two hours, and then lower the temperature to room temperature at a temperature decrease rate of 10 °C/min; then, after the temperature decrease, grind them well for use. It was protonated by dilute hydrochloric acid [19]. Take 1 g of the above ground sample and stir vigorously for 1 h at room temperature in 25 mL of 10 mol/L hydrochloric acid solution, then repeatedly filter with deionized water to remove the excess hydrochloric acid. After vacuum drying, the sample was ground for 30 min and then used. The Ag@Au TNPs/P–CN was synthesized as follows: Take 50 mg of the prepared P–CN disperse ultrasonically in 100 mL of deionized water for 5 h. Then, centrifuge at 3000 r/min for 5 min to obtain a thin layer of P–CN, then diluting 1.25 mL, 2.5 mL, 5 mL, and 7.5 mL of the prepared Ag@Au TNPs with ultrapure water to 10 mL separately. Add the centrifuged thin layer of P–CN, and ultrasonically disperse for 30 min to obtain suspensions of different color depths. After freeze drying, the samples were named as Ag@Au_1.25_/P–CN, Ag@Au_2.5_/P–CN, Ag@Au_5_/P–CN, and Ag@Au_7.5_/P–CN, respectively.

### 2.4. Sample Characterizations

X-ray diffraction (XRD) measurements were performed on a SmartLab XRD spectrometer (Rigaku) with Cu Kα radiation in range of 10~70° (2θ). Transmission electron microscopy (TEM) were taken with a JEM-2100 high-resolution transmission electron microscope. UV-vis diffuse reflectance spectra (DRS) were obtained by a UV-vis spectrophotometer (UV-3600, Shimadzu) with an integrating sphere attachment. PL spectra were measured at room temperature on a Shimadzu RF-5301 fluorescence spectrophotometer with 385 nm excitation wavelength.

### 2.5. Photocatalytic Activity Test

The typical photocatalytic experiment was carried in a photocatalytic hydrogen evolution system (Perfect light, Beijing Lab Solar IIIAG) at 25 °C using a 300 W Xe lamp. Catalyst (10 mg) was dispersed in 40 mL H_2_O and 10 mL methanol; then, we added 100 µL (1 mg/L) H_2_PtCl_6_, and the mixture was stirred under the simulated solar irradiation in vacuum for 30 min. Hydrogen evolution was detected by an online gas chromatograph (GC7900, Tian Mei, Shanghai, TCD with nitrogen as a carrier gas and a 5 Å molecular sieve column) was observed merely under simulated solar irradiation.

### 2.6. Photoelectrochemical Test

Photoelectrochemical analyses were performed using (CHI760E Shanghai Chenhua) a standard three-electrode system. A Pt sheet was used as the counter electrode and a saturated Ag/AgCl (0.1976 V vs. RHE) was employed as the reference electrode. To prepare the working electrode, 1 mg of photocatalyst was uniformly dispersed in a solution containing deionized water (375 μL), ethanol (125 μL), and Nafion (30 μL), and ultrasonicated for 20 min. The resulting colloid was dropped onto an FTO substrate with an area of 1 cm^2^ and then dried at 60 °C for 1 h. The electrolyte was 0.5 M Na_2_SO_4_ solution.

## 3. Results

**Figure 1:**
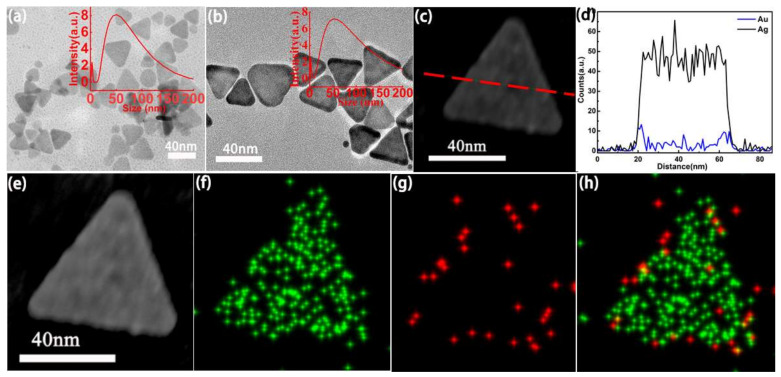
(**a**) TEM of Ag TNPs. Inset in the top-right corner shows the Ag TNPs size histograms. (**b**) TEM of Ag@Au TNPs. Inset in top-right corner shows the Ag@Au TNPs size histograms. (**c**) Scanning transmission image (STEM) of Ag@Au TNPs: a single Ag TNP coated with a thin layer of Au, (**d**) EDS elemental line scan taken along the red dotted line in d to demonstrate the elemental composition of the core and shell of the TNPs. (**e**) STEM image of Ag@Au TNPs and the distribution of Ag (**f**) and Au(**g**) on the Ag@Au TNPs respectively, and the distribution of Ag and Au elements (**h**) in the triangular nanoprisms.

The geometric characterizations of Ag TNPs and Ag@Au TNPs were illustrated in Figure 1. The transmission electron micrograph (TEM) of Ag TNPs are shown in Figure 1a. It can be seen that the Ag TNPs are regular, the tips are obvious, and its average side length is about 50 nm (see Figure 1a). Figure 1b demonstrates that the nanostructures were produced in high yields, the tip part is completely preserved with a perfect triangle, and its average side length is larger than 50 nm. An energy-dispersive X-ray spectroscopy (EDS) elemental line scan in Figure 1d (across a single representative nanoparticle in Figure 1c along with the red dotted line) shows that Au atoms completely surround the Ag TNPs. The normalized mass percentage of Au is 2.89%. The EDS elemental mapping of Ag and Au were performed on the obtained Ag@Au TNPs; as shown in Figure 1e–h, it can be clearly seen that the Au nanoparticles were uniformly deposited on the Ag TNPs. These observations proved that the core–shell structure of the Ag–Au TNPs was prepared successfully.

**Scheme 1:**
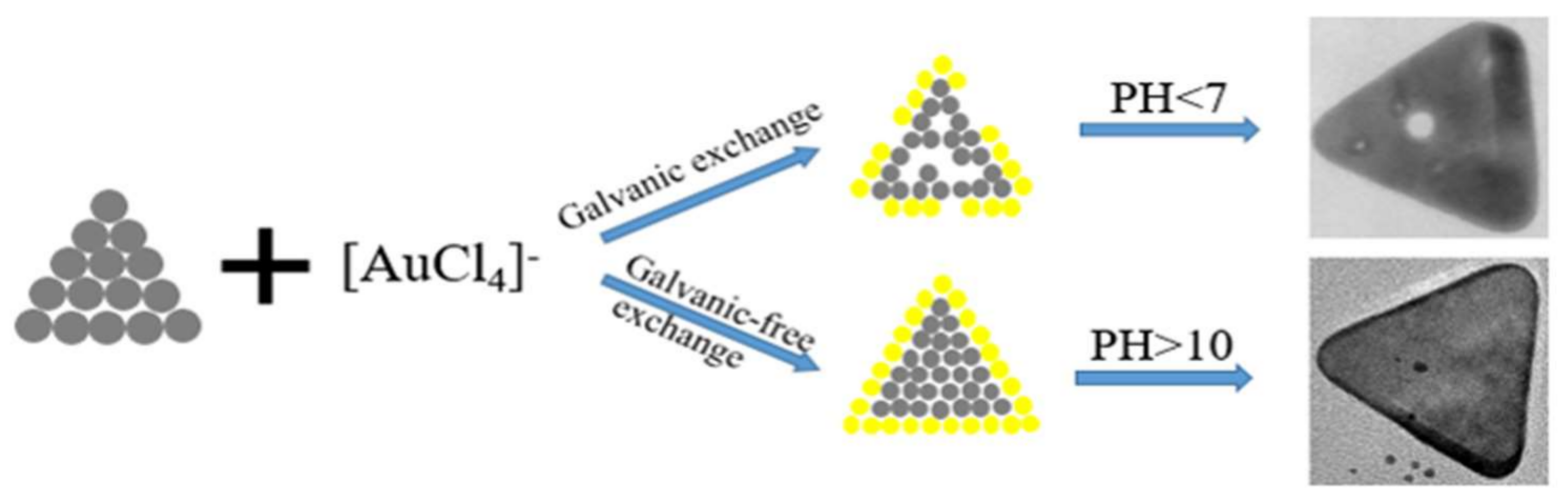
The schematic illustration of the preparation of triangular Ag@Au nanoprisms.

The preparation route of Ag@Au TNPs is shown in Scheme 1. A galvanic-free replacement method was adopted to deposit Au on the surface of the Ag TNPs in this study. When the HAuCl_4_ solution was introduced into triangular silver nanoprisms solution, the system will undergo two kinds of chemical reaction in this synthesis: one is reductant HAuCl_4_ reductive into Au atoms, which can lead to Au depositing on the Ag triangular nanoprisms, forming a dense golden shell. The other is a HAuCl_4_ that directly reacts with Ag atoms in the Ag triangular nanoprisms. It will cause holes in the nanoprisms [20], and the loss of Ag atoms will lead to a weakening of the LSPR effect. When enough NaOH was introduced into the system, the reducibility of the reducing agent ascorbic acid can be improved to enhance the first chemical reaction. At the same time, high pH can weaken the reducibility of HAuCl_4_ solution and inhibit the occurrence of galvanic exchange reaction. In order to prevent the self-nucleation of Au atoms, the HAuCl_4_ solution was dropped into the Ag nanoprisms solution at a rate of 20 µL/min.

**Figure 2:**
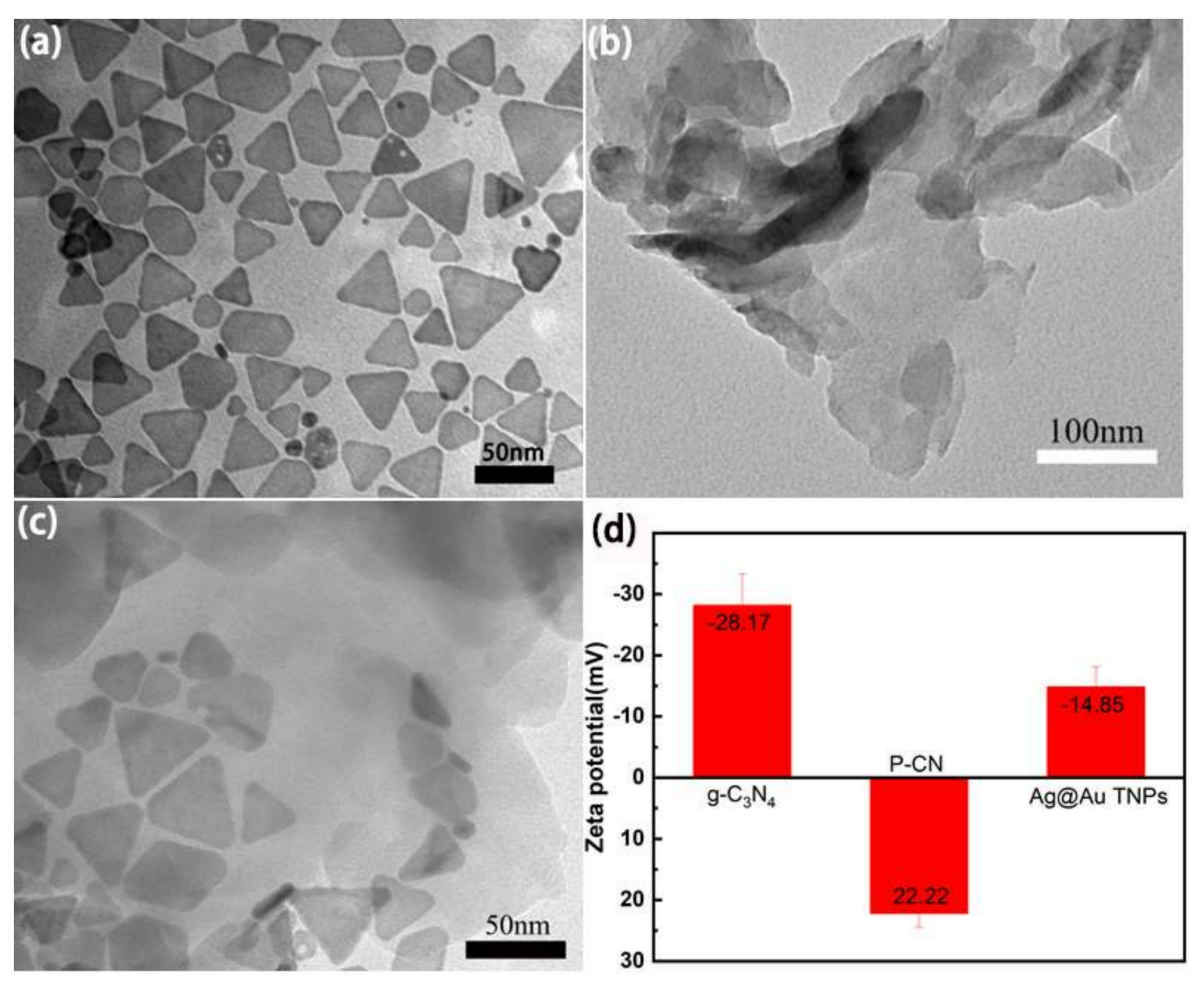
(**a**) TEM of Ag@Au TNPs stored in air condition after 180 days. TEM of P–CN (**b**) and Ag@Au TNPs/P–CN (**c**,**d**) zeta potentials of g-C_3_N_4_, P–CN, and Ag@Au TNPs.

The TEM of Ag@Au TNPs after storing in air condition for 180 days evaluated the morphology stability of the Ag@Au TNPs, as illustrated in Figure 2a. The triangles were still retained, which proved that the Ag@Au TNPs deposited with Au atoms have good stability. The results proved the Ag@Au TNPs were prepared successfully.

In order to test the plasmon enhancement effect of the Ag@Au TNPs on the photocatalytic hydrogen production, a two-dimensional semiconductor material g-C_3_N_4_ was selected as the main catalyst of the photocatalytic system, and the surface charge property of g-C_3_N_4_ and Ag@Au TNPs was also confirmed by zeta potential (Figure 2d). It was observed that the potential of the intrinsic g-C_3_N_4_ is −28.17 mV, while the potential of Ag@Au TNPs is −14.85 mV. Thus, protonating g-C_3_N_4_ makes it positively charged and realizes the binding with Ag@Au TNPs. Not surprisingly, the zeta potential of P–CN is 22.22 mV, so the electrostatic self-assembly method can be used to assemble the Ag@Au TNPs to the two-dimensional P–CN nanosheets. A high-temperature annealing method was used to prepare the intrinsic g-C_3_N_4_. The intrinsic g-C_3_N_4_ was further protonated and subjected to ultrasonic dispersion to obtain ultrathin g-C_3_N_4_ nanosheets, as shown in Figure 2b. The morphology of loaded with Ag@Au TNPs is shown in Figure 2c, and it is found that the Ag@Au TNPs are uniformly dispersed on the P–CN nanosheets.

**Figure 3:**
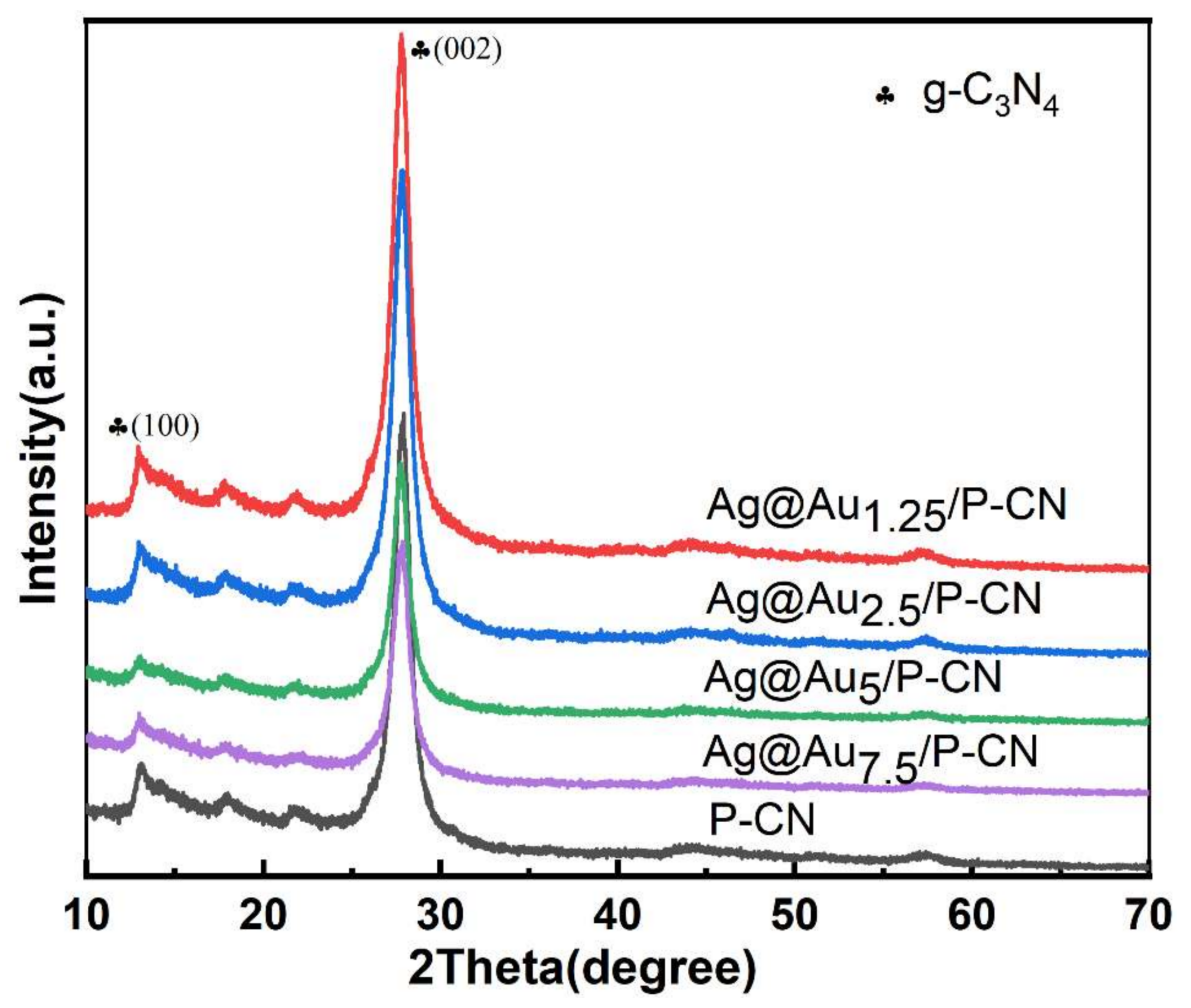
XRD of P-CN and Ag@Au_1.25_/P–CN, Ag@Au_2.5_/P–CN, Ag@Au_5_/P–CN, and Ag@Au_7.5_/P–CN.

The XRD test was performed to explore the influence on the crystal structure of P–CN after introducing the triangular Ag@Au nanoprisms, as shown in Figure 3. It can be seen that 13.1° and 27.4° corresponds to the (100) and (002) crystal plane of g-C_3_N_4_, respectively. The diffraction peak of g-C_3_N_4_ is completely retained for the samples with different loading amounts, which proves that the introduction of the Ag@Au TNPs structure does not damage the crystal structure of P–CN. In addition, the diffraction peaks of Ag and Au were not observed in different samples, which may be due to the low loading content and high dispersity. We did not find any extra Ag–Au alloy peaks.

**Figure 4:**
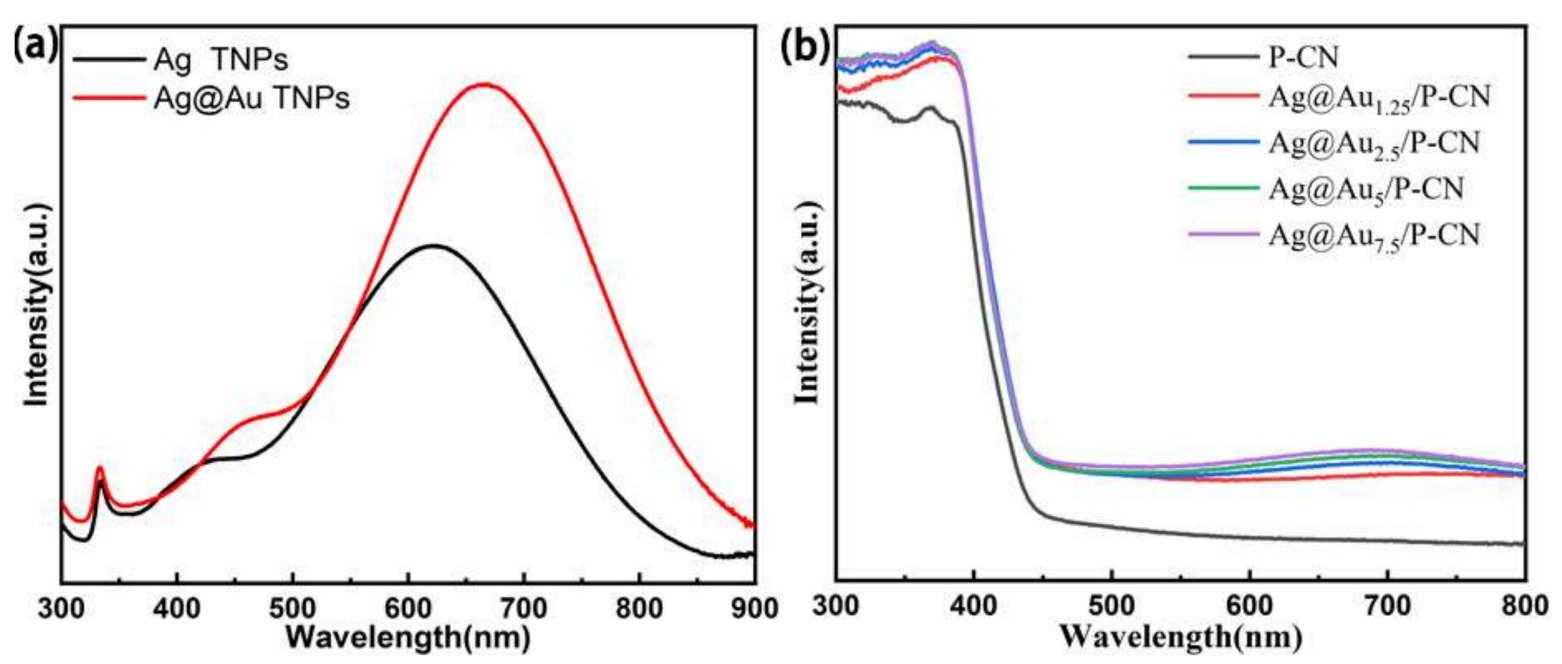
(**a**) UV-vis absorption spectra of Ag TNPs and Ag@Au TNPs. (**b**) UV-vis absorption spectra of the system with P–CN and Ag@Au_1.25_/P–CN, Ag@Au_2.5_/P–CN, Ag@Au_5_/P–CN, Ag@Au_7.5_/P–CN.

The light-harvesting characters of Ag TNPs, Ag@Au TNPs, P–CN, and Ag@Au TNPs/P–CN were measured by UV-vis absorption spectra in Figure 4. Figure 4a shows the UV-vis absorption spectra of Ag TNPs and Ag@Au TNPs. Compared to the LSPR peak position of the Ag TNPs, the peak of the Ag@Au TNPs has a slight red shift from 650 to 670 nm. This was attributed to the deposition of Au on the Ag TNPs, resulting in a larger side, which further proves that the bimetallic Ag@Au TNPs were successfully synthesized. Then, the UV-vis test was further carried out, and as shown in Figure 4b, the absorbance of the system after loading Ag@Au TNPs increased significantly in the visible light region. The absorption spectrum of the Ag@Au TNPs shows a significant enhancement near 650 nm, which further indicates that the Ag@Au TNPs were successfully loaded onto the protonated g-C_3_N_4_ nanoprisms, which can increase the light absorption intensity of the system gradually. This is due to the strong light absorption capacity of Ag@Au TNPs. This proves that Ag@Au TNPs can synergistically increase the absorbance of g-C_3_N_4_ nanoprisms.

**Figure 5:**
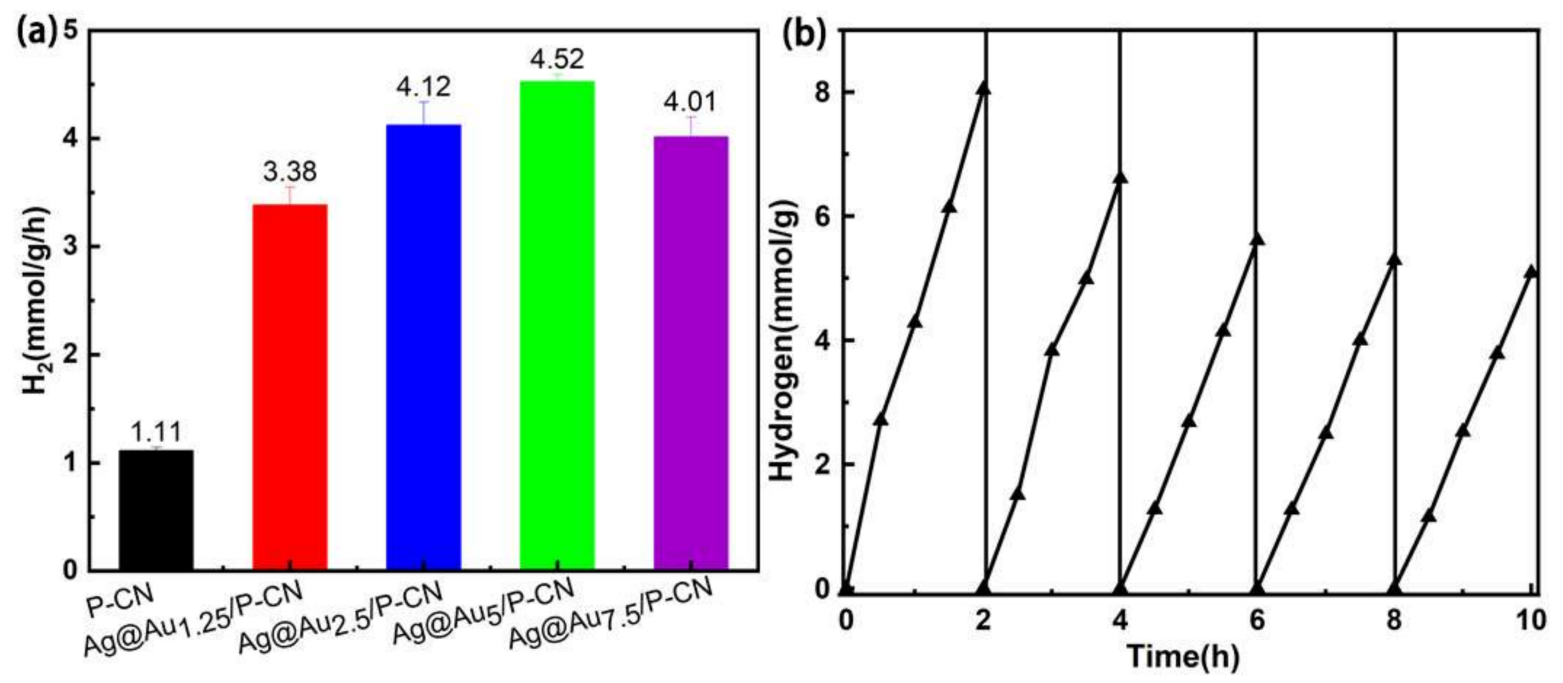
(**a**) The hydrogen production of P–CN and Ag@Au_1.25_/P–CN, Ag@Au_2.5_/P–CN, Ag@Au_5_/P–CN, Ag@Au_7.5_/P–CN and (**b**) Cycle stability test of the Ag@Au_5_/P–CN.

To elucidate the effect of the Ag@Au TNPs on the photocatalytic H_2_ production reaction activity of the Ag@Au TNPs/P–CN composite, P–CN and Ag@Au TNPs/P–CN photocatalysts are measured under simulated solar light irradiation by using a 300 W Xenon lamp with an AM 1.5G cutoff filter, respectively. Methanol was used as the sacrificial reagent to consume the plasmon-induced holes in TNPs. From Figure 5a, the hydrogen production of the system after loading Ag@Au TNPs had been greatly improved, the photocatalytic hydrogen production effect gradually increased with the increase in Ag@Au TNPs loading amounts, and the hydrogen production rate reached the maximum at Ag@Au_5_/P–CN: the hydrogen production of Ag@Au_5_/P–CN was up to 4.52 mmol/g/h, which was the hydrogen production of P–CN nanosheets (1.11 mmol/g/h) 4.1-fold. As the loading amount continues to increase, the hydrogen production efficiency decreases, which may be due to the nanoparticle agglomeration that reduces the light absorption efficiency. In order to explore the stability of the sample during use, it was tested for five consecutive cycles of hydrogen production in ten hours. It was found that after the third test, the hydrogen production of the sample dropped by 25%. Then, it gradually stabilized, which may be due to the gradual consumption of the sacrificial agent in the system.

**Figure 6:**
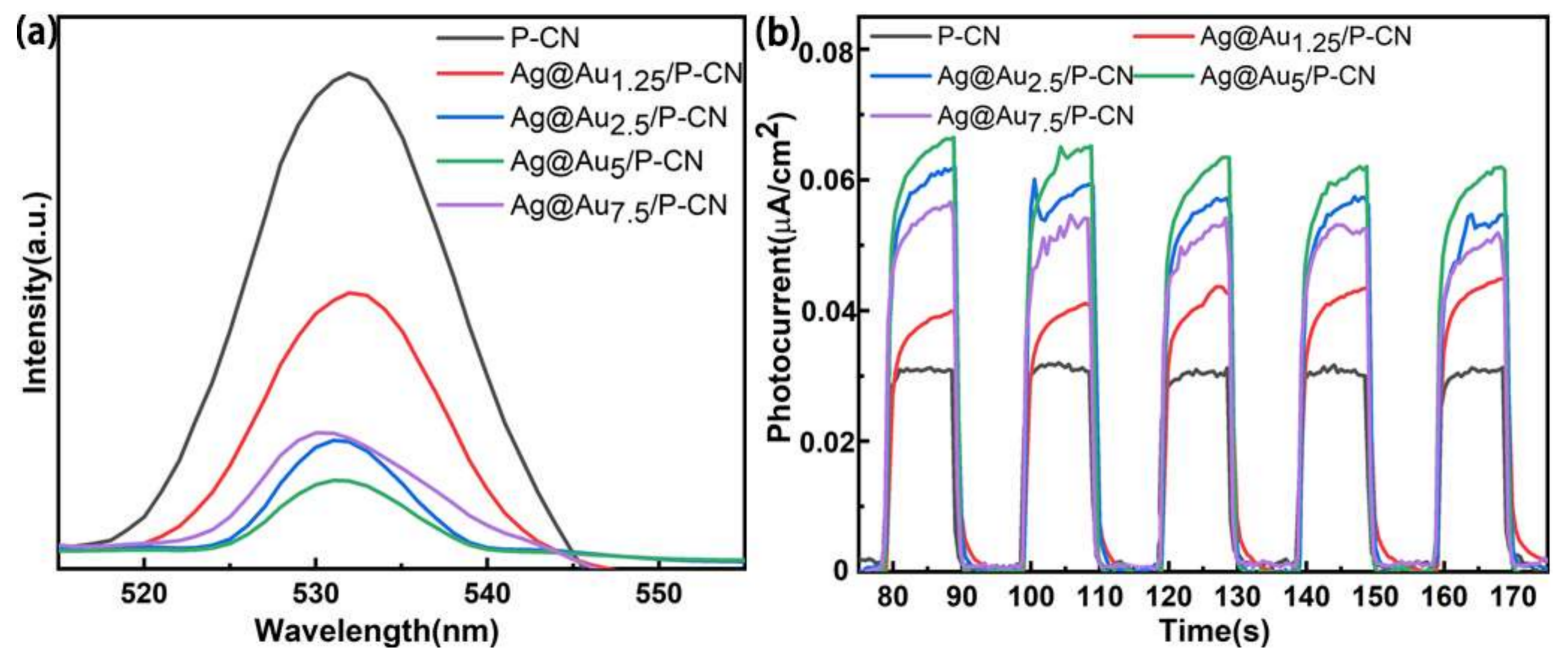
(**a**) PL spectrum of P-CN and Ag@Au_1.25_/P–CN, Ag@Au_2.5_/P–CN, Ag@Au_5_/P–CN, and Ag@Au_7.5_/P–CN. (**b**) The photocurrent of the system with P-CN and Ag@Au_1.25_/P–CN, Ag@Au_2.5_/P–CN, Ag@Au_5_/P–CN, and Ag@Au_7.5_/P–CN.

The photoluminescence (PL) spectra and photocurrent tests were further carried out in Figure 6 to study the enhancement of H_2_ production in depth. As observed in Figure 6a, the PL intensity of the loaded Ag@Au TNPs is significantly lower than that of the pure P–CN, and the PL intensity of Ag@Au_5_/P–CN reaches the lowest. On the one hand, the well-preserved tips of the Ag@Au TNPs generates a strong electromagnetic field under the irradiation of simulated solar light, which inhibits the recombination of photogenerated electron–hole pairs in the P–CN nanoprisms and prolongs the life of photogenerated charge carriers. On the other hand, the Schottky junction formed between the Ag@Au TNPs and P–CN promotes the capture of the hot electron, which indicates that the Ag@Au TNPs can effectively extend the carrier life and make it a better role in photocatalytic hydrogen production. The photocurrent test results of the sample in Figure 6b show that the photocurrent of the Ag@Au_5_/P–CN reaches 0.07 µA/cm^2^, which is 2.3 times more than P–CN; this indicates that the load of Ag@Au TNPs increases the amount of photogenerated charge carriers, and the result is in accordance in the PL spectral test. So, the increase in H_2_ production is the result of the synergistic effect of electromagnetic field enhancement at the tips of the Ag@Au TNPs and the chemical enhancement.

**Figure 7:**
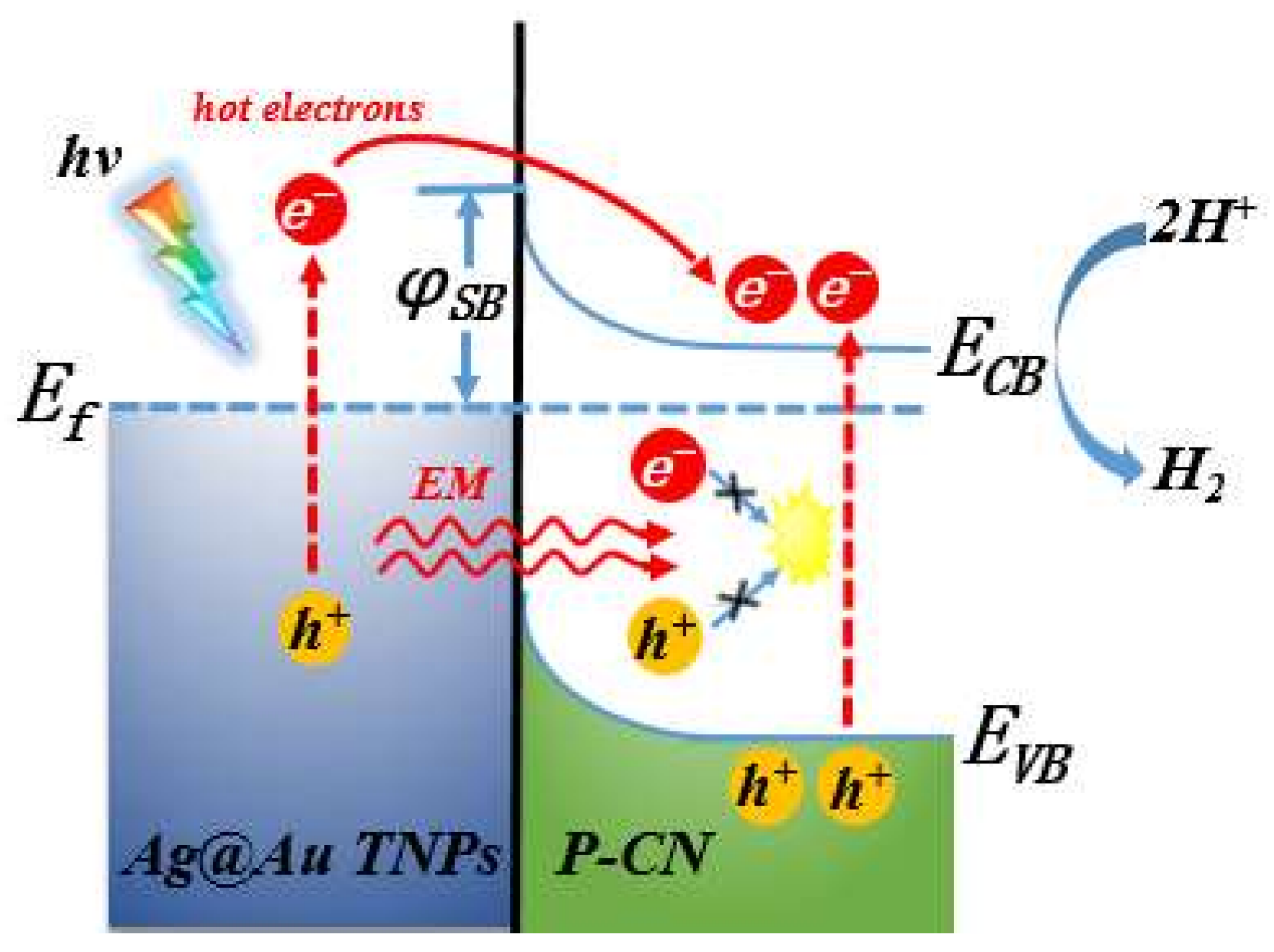
The proposed photocatalytic mechanism of Ag@Au TNPs/P–CN for H_2_ evolution.

Based on the above results, a possible mechanism is proposed, as shown in Figure 7. The P–CN contacts with the Ag@Au TNPs will form a Schottky junction. Under the simulated solar irradiation, the Ag@Au nanoprisms generate hot electrons by LSPR. The Schottky barrier helps to trap the transferred hot electrons in the conduction band of the P–CN by delaying electron transmission back to the Ag@Au TNPs [21]. Meanwhile, the hot electrons gathered together in the tips of the Ag@Au TNPs to generate the electromagnetic field. It can be used to inhibit the recombination of electron–hole pairs produced on P–CN [6]. The hot electron effect and electromagnetic field enhanced the photocatalytic hydrogen production efficiency of the system simultaneously.

## 4. Discussion

In this paper, Ag TNPs with stable morphology and uniform dispersion were pre-pared by a chemical reduction method, and the Ag@Au TNPs were synthesized by using the effect of significantly enhancing the reducibility of ascorbic acid under higher pH conditions to inhibit galvanic exchange. The Ag@Au TNPs/P–CN plasmonic photocatalysts with different loadings were obtained by electrostatic self-assembly. The well-preserved tips of the Ag@Au TNPs can effectively generate an electromagnetic field to suppress the photogenerated electron–hole pairs recombination on the semiconductor. The Schottky junction between the Ag@Au TNPs and P–CN also helps the hot electrons trap P–CN for hydrogen production reaction under the irradiation of simulated sunlight. The Ag@Au TNPs retains the good plasmon effect of the silver TNPs while increasing their stability so as to better play roles in actual production in life and some other applications.
